# Usability and Usefulness of a Mobile Health App for Pregnancy-Related Work Advice: Mixed-Methods Approach

**DOI:** 10.2196/11442

**Published:** 2019-05-09

**Authors:** Monique van Beukering, Adeline Velu, Liesbeth van den Berg, Marjolein Kok, Ben Willem Mol, Monique Frings-Dresen, Robert de Leeuw, Joris van der Post, Linda Peute

**Affiliations:** 1 Department of Obstetrics and Gynecology Amsterdam UMC University of Amsterdam Amsterdam Netherlands; 2 Department of Medical Informatics Amsterdam UMC University of Amsterdam Amsterdam Netherlands; 3 Department of Obstetrics and Gynaecology Monash Medical Centre Monash University Melbourne Australia; 4 Coronel Institute of Occupational Health Amsterdam Public Health Research Institute, Amsterdam UMC University of Amsterdam Amsterdam Netherlands; 5 Department of Obstetrics and Gynecology Amsterdam UMC Vrije Universiteit Amsterdam Amsterdam Netherlands; 6 Department of Medical Informatics Center for Human Factors Engineering of Health Information Technology, Amsterdam UMC University of Amsterdam Amsterdam Netherlands

**Keywords:** mHealth, eHealth, mobile phone, pregnancy, work, occupation, occupational exposure, qualitative research

## Abstract

**Background:**

Pregnant women are often unaware of the potential risks that working conditions can cause to them and their unborn child. A mobile health (mHealth) app, the *Pregnancy and Work* (P and W) app, developed by a multidisciplinary team and based on an evidence-based guideline for occupational physicians, aims to provide advice on work adjustment during pregnancy.

**Objective:**

This study evaluates the usability of the mHealth P and W app and the perceived usefulness of the *work advice*, the main goal of the app, by potential end users.

**Methods:**

A total of 12 working pregnant women participated in think aloud usability sessions and performed 9 tasks. All think aloud sessions were recorded, transcribed, and coanalyzed. The usability problems were rated for their severity in accordance with Nielsen severity scale. The completion rates and time taken for completion of tasks were registered. In addition, participants were questioned on demographics and user characteristics and were asked to evaluate the value of the app by filling in the Intrinsic Motivation Inventory (IMI) score and the System Usability Scale (SUS) questionnaire.

**Results:**

In total, 82 usability problems with a severity ≥1 were identified, of which 40 had severity ≥3. The main usability problems concerned the interpretation of terminology used in the app’s questionnaires and difficulties in finding and understanding the work advice. Furthermore, 10 out of 12 participants were able to open the work advice page in the app. Only 7 out of these 10 participants understood and intended to follow the work advice. The overall mean IMI score was relatively high (5 out of 7), indicating that the participants did indeed value the use of the app. This IMI score corresponded to the overall mean SUS score (68 out of 100) and the mean grade given to the P and W app (7 out of 10).

**Conclusions:**

This think aloud usability study showed that the information provided in the P and W app was considered valuable by the end users, working pregnant women, and it meets their needs; however, usability issues severely impacted the perceived usefulness of the work advice given in the app.

## Introduction

### Background

Many women continue to work during their pregnancy. In the United States, more than 65% of pregnant women work, whereas in the Netherlands, around 80% [1,2]. During pregnancy, exposure to certain working conditions, such as physically demanding work, long working hours, working in night shifts, and stress, are associated with preterm birth, low birth weight, and fetal abnormalities [3-12]. As pregnant women are often not aware of these risks, they do not adjust their working conditions [13].

Mobile health (mHealth) apps can offer a suitable solution to this problem as women of reproductive age who are expecting a child are frequent consumers of Web-based health information [14-17]. mHealth, defined as the use of mobile devices for medical and public health practice [18], could therefore inform pregnant working women about work-related pregnancy risks, to increase their awareness of these risks and their associated need for change in working conditions.

However, evidence on the effectiveness of mHealth apps in general is limited [17,19]. Prior studies provide little information as to how best to design them [20-24]. Adequate consideration of the needs of their intended users is necessary so that they are easy to use and perceived as useful [25,26]. *The extent to which a product can be used by specified users to achieve specified goals with effectiveness, efficiency, and satisfaction in a specified context of use* is the definition for applied usability, based on the International Standardization Organization [27]. To assess and improve upon the usability of mHealth apps, a wide range of usability evaluation methods (UEMs) is available to detect problems in the user-system interaction. UEMs thus assess human interaction with a system for the purpose of identifying those facets of this interaction which can be improved [28]. Ideally, the design process of any health-related product is conducted in an iterative fashion to better fit with the end user population. Utilizing UEMs in such an iterative design process in the health care domain is especially important as the poor design and usability of medical products can lead to harmful consequences [29,30]. Therefore, the utilization of UEMs during the development and testing process of health apps is widely recommended throughout research [31,32].

In this study, we developed an mHealth solution (the Pregnancy and Work [P and W]) app that aimed to provide information and advice about work-related pregnancy risks [33]. With this advice, pregnant women can adjust their work. The P and W app content is based on the evidence-based guideline for occupational physicians, *pregnancy, postpartum period, and work* [34]. In a prior study, the results of 2 multidisciplinary focus group meetings provided content and design instructions for the development of the P and W app [35].

### Objectives

Think aloud (TA), an UEM method, was chosen in this study to assess the usability of the P and W app with potential end users to reveal cognitive processes in the app’s user interaction that result in user-interaction problems. The TA method requires participants to talk aloud (ie, verbalize their thoughts) while performing or solving a task to reveal their cognitive processes while interacting with the app, which may result in user-interaction problems [36-38]. In this way, the TA helps to understand how pregnant woman think—or believe they think—the P and W app works (ie, their mental model) [38]. Mismatches in the end users’ mental model of an app and the app’s design can severely influence its usability and subsequently its use in practice. This study therefore evaluated the usability of the P and W app and also how potential end users experienced the usefulness of the work advice; this was the main goal of the app.

## Methods

### Participant Recruitment

A total of 2 obstetric care facilities, representing a broad variety of patient groups, participated in this study. Posters and flyers were distributed in both locations. The inclusion criteria were drawn up by an obstetrician and occupational physician. If patients met the inclusion criteria, they were invited to participate in the study. The inclusion criteria were Dutch working women, who were less than 20 weeks pregnant. The criterion of being less than 20 weeks pregnant was deliberately stated as the work advice for pregnant women under 20 weeks of pregnancy can be different than that for those after 20 weeks of pregnancy. Eligible participants were recruited in the waiting area of the physician’s office. Recruitment of participants continued until a total of 12 female patients agreed to participate in the TA sessions and evaluate the app; this was the first time they used the P and W app. All participants included in the study were offered a gift card worth €15. An app for this research was submitted to the ethical board of the Amsterdam University Medical Center. The board confirmed that the Medical Research Involving Human Subjects Act did not apply to this study. All data from the 12 participants were anonymously processed. Informed consent was obtained from all participants, allowing us to use the data for analysis.

### P and W Pregnancy and Work App and Study Flow

The P and W app (Dutch and English) was created as a Web-based app, accessible from every type of mobile browser, with an adaptive design for desktop and mobile phone use.

**Figure 1:**
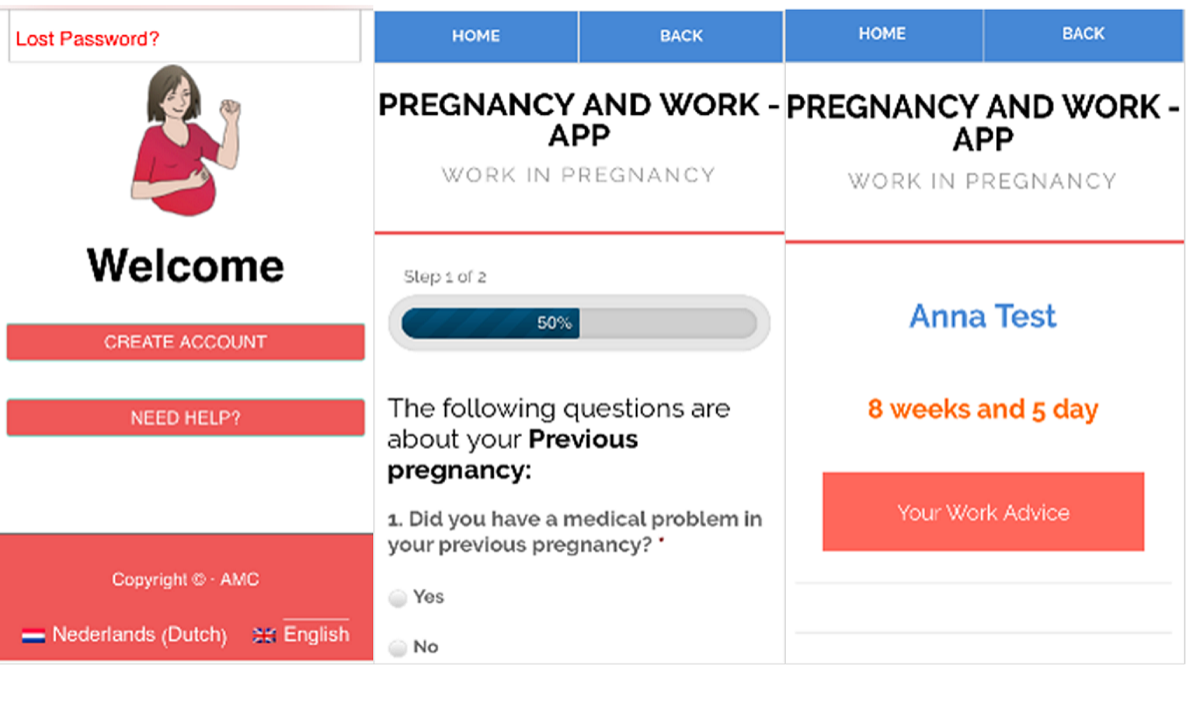
Examples of screenshots of Pregnancy and Work App: the Welcome page, the Questionnaire page, and the Work Advice page.

The P and W app requires the user to create an account to gain access to its content. After creating an account, a user needs to complete a questionnaire about her pregnancy-related medical and work conditions (Figure 1). When completing this questionnaire, the user will be directed to the home page of the app, from where she can navigate to all other pages. On the home page, users can view monthly pregnancy- and work-related advice messages, which are also sent by email. In addition, the app provides messages about the growth of the unborn baby as the weeks pass. Next to the baby messages, a video with tips and information about pregnancy-related work advice can be viewed on the home page. Participants were given access to a Dutch beta test version of the P and W app.

### Phase I: Preparation

**Figure 2:**
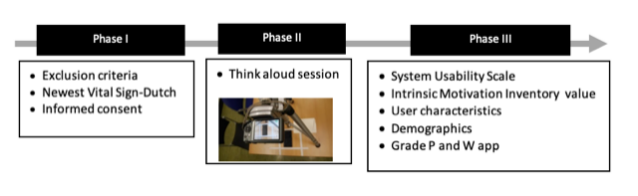
Overview of study setup.

Participants were informed about how the TA session would be performed; see Figure 2 for the full study setup. After a 2-week reflection period, a condition for participation in the research, an appointment was made with those women who wanted to participate in the study. The TA session then took place at their next visit (follow-up consultation) to the obstetrics department. After signing an informed consent form, the participant completed a short survey, the validated health literacy (HL) assessment tool— *the Newest Vital Sign*, translated to Dutch—to analyze its potential influence on the TA outcomes (Stage I, Multimedia Appendix 1 [39,40].

### Phase II: Think Aloud Usability Testing

**Figure 3:**
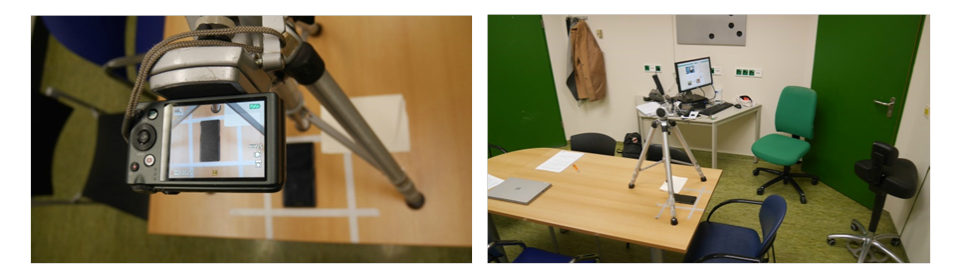
Think aloud session set-up.

Participants started with practice tasks on how to *think aloud* (Multimedia Appendix 2). Each participant was informed that the researcher (LvdB) was solely interested in the app’s performance and would only interrupt the participant to provide new tasks and to encourage her to keep talking to break silences longer than 5 seconds [41]. A participant had to complete 9 tasks in total that were centered around the core purpose of the app (Multimedia Appendix 3). Tasks were developed in collaboration with the developer and project supervisors of the P and W project. All TA sessions were recorded via video camera. Voice and screen (of their mobile phone) were also recorded (Figure 3).

### Phase III: Usability and Motivation Questionnaires

After the TA test was finished, the SUS survey was given to the participant to assess the perceived usability of the P and W app [42] (Multimedia Appendix 4). The SUS comprises 10 statements which the participant had to rate on a scale from 1 (strongly disagree) to 5 (strongly agree) to indicate the extent to which she agreed. Then, a short survey selection of the Intrinsic Motivation Inventory (IMI) was given to assess a self-reported evaluation on how much the participant valued the P and W app [43] (Multimedia Appendix 4). The IMI value subscale comprised 7 statements which the participant had to rate on a scale from 1 (do not agree) to 7 (strongly agree) to indicate the extent to which she agreed. An additional short survey was developed to gain more insight into participants’ demographics, medical history related to pregnancy, prior experience with (pregnancy-related) mobile apps, and working hours (Multimedia Appendix 4). We asked all participants whether they had received and would follow the work advice (Multimedia Appendix 4). Finally, participants were asked to give the P and W app a grade on a scale from 1 to 10, where 1 was the lowest and 10 the highest grade.

### Data Collection and Analysis

The TA sessions were videotaped, reviewed multiple times, and transcribed to verbal protocols by 2 researchers (LvdB and LP). To gain insight into the effectiveness and efficiency of the participants in performing tasks, each TA session transcription comprised text spoken by the participant and included task completion time stamps and time taken for task completion. To analyze the usability problems participants encountered in more detail, we performed a thematic analysis for which a coding scheme was developed bottom-up in 3 iterative cycles as described by Jaspers [44]. We analyzed 2 TA sessions in-depth to develop a raw coding scheme (first cycle). Usability issues encountered by participants were then given a specific description. We subsequently discussed the resulting codes and grouped them to determine the main themes in the data (second cycle). The developed coding scheme was then applied to code and analyze all verbalizations, this was performed by LvdB and checked by LP. All new issues were discussed to determine whether they were within the branches of the coding tree or if a new main theme had emerged. Usability problems were rated on severity in accordance with Nielsen severity scale [45]. Nielsen severity scale is a rating scale from 0 to 4 (Textbox 1), that allows for the prioritization of usability problems that need to be revised in the development process. The questionnaires were completed on paper and put in a database for data analysis.

All data filled in by the participants in the P and W app during the TA sessions were specifically transcribed into a different file to test for task efficacy in relation to the IMI-given work advice by the system. Verbalizations of task 6 in the TA sessions (*find the work advice*) were assessed to analyze whether participants would follow the work advice. These results were compared with the results of the IMI on participant level and the questions about the work advice from questionnaire 3 (Multimedia Appendix 4). Finally, the SUS was used to assess the perceived usability of the P and W app.

**Nielsen’s severity scale**

0–I do not agree that this is a usability problem at all.

1–Cosmetic problem only: need not to be fixed unless extra time is available on project.

2–Minor usability problem: fixing this should be given low priority.

3–Major usability problem: important to fix, so should be given high priority.

4–Usability catastrophe: imperative to fix this before product can be released.

## Results

### Participant Characteristics

The TA sessions with the participants (N=12) took place between April and June 2017. Most participants scored high (=adequate) HL. All participants had paid jobs and used a mobile phone. The average gestational age of the participants was 15 weeks and 50% (6/12) of the participants were pregnant for the first time (Table 1).

### Task Completion

The effectiveness and efficiency of the participants in performing tasks were measured by completion rates and times and the usability problems. The completion rates and times can be found in Table 2. The average duration of a TA session was 19 min 55 seconds (SD 5 min 25 seconds). Task 1, *create an account*, had a much higher completion time than the other tasks. Tasks 2, 3, 5, and 9 were completed by all participants. Tasks 1, 4, 6, 7, and 8 were not completed by all participants. The first 3 tasks took, on average, the longest time to complete, ranging from 1.5 min to 4 min. Task 9 had the fastest mean completion rate of 4 seconds.

### Usability Problems

The TA study identified a total of 101 usability issues, 82 of which were considered *real* usability problems (ie, severity ≥1), whereas 40 usability problems were rated with a severity of 3 (major) or 4 (catastrophic). In addition, the participants encountered 11 unique bugs when using the P and W app. An overview of the most severe usability problems can be found in Table 3. The high completion time with *create an account* (Table 1) seemed to have a connection with the many usability problems in this area (Table 3). None of the participants experienced (severe) usability problems when completing tasks 5, 7, and 9. In the following section, we give an in-depth analysis of the severe usability problems detected regarding *terminology interpretation* and *finding and understanding the work advice* that directly impacted the participants’ perceived usefulness of the advice given in the app.

**Table 1:** Participants’ basic demographics and characteristics (N=12).

Characteristics		Statistics
Age (years), mean (SD)		33 (3.8)
**Education** **(secondary school), n**		
	Higher education	8
	Intermediate vocational education	4
**Health literacy, n**		
	High	11
	Low	1
Paid job, n		12
Working time (hours per week), mean (SD)		37 (6.15)
Gestational age (weeks), mean (SD)		15 (3)
Previous pregnancy, n		6
Children, n		5
**Mobile phone (operating system), n**		
	Android	7
	iPhone	5

**Table 2:** Completion rates and time taken per task (N=9) by participants.

Task	Completion rate	Time taken for completion (seconds), mean (SD)
1. Create an account	10/12	240 (83)
2. Fill in a questionnaire	12/12	179 (101)
3. Adjust answers to the questionnaire	12/12	96 (74)^a^
4. Find *your rights and tips for consultation* page	11/12	31 (38)
5. Find *baby message(s)*	12/12	16 (10)
6. Find the *your work advice* page	10/12	10 (8)
7. Find the *print/save* button	10/12	9 (9)
8. Find the goal of the Pregnancy and Work P and W app	11/12	32 (18)
9. Log out of the app	12/12	4 (4)

^a^A total of 2 participants initially did not understand this task.

**Table 3:** Overview of severe usability problems per main problem type.

Usability problem^a^	Frequency	Severity	Source of main problem
Unclear buttons	12	2 to 4	Create account
Functionality with layout	11	4	Create account/home page
Terminology interpretation problems	8	4	Create account/home page
Finding and understanding work advice	8	4	Home page/work advice

^a^Multimedia Appendix 5 shows an overview of all the usability problems.

### Qualitative Assessment

#### Terminology Interpretation Problems

Participants had to complete a questionnaire about their pregnancy-related medical conditions, previous pregnancy (if relevant), and work conditions using the app. Several terminology interpretation problems arose during the TA study, which consequently prevented the participants from receiving accurate personal work advice. For example, when asked whether problems had been experienced during the previous pregnancy, participants were unsure whether *previous pregnancy* implied the immediate previous pregnancy or also the pregnancies before that. One participant who had not experienced problems during her previous pregnancy, but did experience issues during the pregnancy before that, assumed it implied her direct previous pregnancy. Her confusion in answering the question correctly affected the outcome of the work advice, as relevant information was missing:

Okay. Um. “Did you have a medical problem in your previous pregnancy?” This is about my last pregnancy, I think, and not the pregnancies before. So, I'm assuming that. And then it's a no.

Participant 5

Problems were also prevalent when, in closed-ended questions, the participant did not find the answer that applied to her within the limited selection of possibilities of medical disorders. When given a list of potential problems during a previous pregnancy, participants experienced troubles in selecting the best suited option to describe their problem:

...But I do not know if that should be put under “deceased child” or “child born before a gestational age of 37 weeks”? You know what I mean?

Participant 3

Another example of a terminology interpretation problem that affected the outcome of the work advice was related to the question of *being exposed to any chemical agents in the work environment*, followed by a list of examples. Several participants did not notice the list of examples and answered *no*. Furthermore, 2 other participants did not know whether an agent that they worked with should be considered chemical, as it was not on the list of examples:

...Yes, with hair dye. Is that chemical?

Participant 9

...I’m having doubts. I work with laughing gas. That’s not very chemical, but...I don’t know whether I should answer yes or no.

Participant 11

#### Finding and Understanding the Work Advice

**Figure 4:**
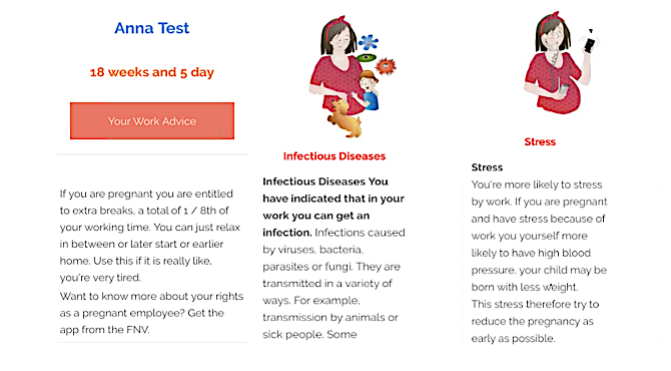
The “Your Work Advice” button on the home page with examples of work advice (infectious diseases and stress) when the button is clicked.

Participants also experienced problems in understanding the work advice because of central design problems in the interface. One of the first issues encountered was that the participants expected the app to show them something different than what it actually did. Participants expected the app to show their work advice directly on the homepage, as they perceived this to be the essential goal of the app. They did not expect to have to search for it in the interface or take any other action to find it. For example, participant 6 did not understand that the *your work advice* button was clickable and therefore sought work advice elsewhere or stated that she could not find it (Figure 4):

...Oh, let's see if that is somewhere. No idea. [Scrolls down and up] Have a look. Here is my work advice. Uh... [Scrolls up and down, multiple times] No, I have no idea.

Participant 6

A different example related to the participants stating that they saw their work advice depicted on the home page. However, the home page only provided a small section with tips and information about pregnancy-related work advice, which some clearly interpreted as the entire personal work advice. A total of 2 participants thought this was the case; therefore, both of them missed the actual content of the *your work advice* page:

I’ve just seen my work advice. [Scrolls up and down. Scrolls to top of the page. Taps the back button. Loads page] Yes, your work advice. I have already read it. So, it is here.

Participant 8

Another usability issue was related to the fixed structure in which the work advice was presented in the mobile interface. Depending on the answers given in the questionnaire, specific information followed on the work advice page. The resulting advice therefore included some sections without advice and some sections with the advice, spread over the mobile interface. One participant did not get work advice below the *work header*; however, she did receive work advice with regard to issues during her previous pregnancy, but this would only have become visible if she had scrolled the page down. She therefore missed the advice given:

None? That’s easy. I don’t need to make any work adjustments. I don’t think so either, because I have an office job.

Participant 1

#### User Evaluation: Intrinsic Motivation Inventory and System Usability Scale

The task efficacy of task 6, *find the “your work advice” page*, was analyzed in relation to the detected usability problems in *finding and understanding the work advice* and combined with the results of the IMI, SUS, and questions about the work advice from questionnaire 3 (Multimedia Appendix 4). Some participants never reached the work advice page on the app (17%) but thought they did, whereas 3 out of 12 participants (25%) were convinced that they had not received this advice (Table 4). However, all participants did actually receive some form of pregnancy-related work advice. Among the 9 participants who stated that they had received work advice, 2 indicated that they would not follow it.

Using the IMI, we assessed the self-reported evaluation of how much the participants valued the P and W app; the overall mean IMI value score was 5 (SD 0.9) out of 7. The perceived usability of the P and W app was stated by the SUS. The overall mean SUS was 68 (SD 11). Finally, the participants were tasked to give the P and W app a grade on a scale from 1 to 10; the mean grade given to the P and W app was a 7 (SD 0.89; Table 4).

**Table 4:** User evaluation based on the use of work advice, Intrinsic Motivation Inventory (IMI), System Usability Scale (SUS), and grade.

Participant number	Did you receive work advice from the app?^a^	If so, do you intend to do something with this work advice?^b^	IMI^c^	SUS^d^	Grade
1	No^e^	N/A^f^	5.57	85	8
2	No	N/A	4.29	77.5	7
3	Yes	Yes	3.71	55	5
4	Yes	Yes	5.00	77.5	7
5	Yes	Yes	5.14	65	8
6	Yes	No	4.43	77.5	6
7	Yes	Yes	5.57	57.5	7
8	Yes	No	3.00	70	7
9	No	N/A	4.29	75	7
10	Yes	Yes	6.29	55	6
11	Yes	Yes	5.29	50	6
12	Yes	Yes	4.86	72.5	6

^a^Multimedia Appendix 4-III Questionnaire 3, Question 1.

^b^Multimedia Appendix 4-III Questionnaire 3, Question 2.

^c^IMI score; 1=*not at all true* to 7=*very true*.

^d^SUS score; 1=*strongly disagree* to 5=*strongly agree*.

^e^Participants 1, 2, and 9 were convinced that they had not received work advice; however, all participants did receive work advice.

^f^N/A: not applicable as the participant indicated that she did not receive work advice.

## Discussion

### Principal Findings

The overall effectiveness and efficiency of the 12 participants in performing tasks in the TA sessions are gauged by the completion times and rates and the usability problems. The TA study identified 82 usability problems with a severity ≥1, of which 40 had severity ≥3. The high completion time of the task to create an account seemed to be connected to the many usability problems that participants experienced in this task. As *creating an account* in an mHealth app is not usually part of the core, there is a chance that the design of this first part of the app may be neglected. Design errors in *creating an account*, however, increase the risk of participants dropping out quickly.

We performed an in-depth analysis of the severe usability problems detected regarding *terminology interpretation* and *finding and understanding the work advice* as these issues directly impacted the usefulness of the app. As participants were unable to correctly interpret the terminology in the questionnaire about previous pregnancies, medical disorders, and chemical agents, they did not understand how to complete the questionnaires corresponding to their personal situation. They thus did not receive the correct personal work advice for their circumstances.

Participants also had a different expectation of what the app would show them. Their mental model, the way information is represented in the mind of the end user, affected how they acted in the system in filtering the relevant information. The mental model of the participants did not match how the designer developed the system, as the designer had based it on his own mental model of how future end users would act on the information presented. The mental model of end users, which encompasses values, beliefs, and knowledge, creates perspectives for filtering information and guiding problem solving [46] and has the ability to affect how a person acts [47], differed from that of the designers. The users therefore also experienced problems with understanding the work advice, as their expectations did not match how the designer developed the system (based on his mental model of how future end users should act on information).

Due to the usability problems in its design, 10 out of 12 participants were able to open the work advice page. Only 7 out of these 10 participants understood and intended to follow the work advice given in the app, which was the main goal of the app.

The overall mean IMI score was relatively high (5 out of 7), indicating that the participants did indeed value the use of the app. This corresponded to the overall mean SUS score (68 out of 100) and the mean grade given to the P and W app (7 out of 10).

### Comparison With Prior Work

Our main results indicated the effect of the app’s navigational structure and screen design on the ability of a specific group of participants—pregnant working women—to find work advice and their intention to follow it thereafter. Other studies in mHealth and electronic health that have applied the TA method have demonstrated that although participants *think* that they have achieved the main goal of using the apps, in reality its intended objective was not reached [48,49]. In one study the researchers observed that the majority of participants, older cancer patients, were not able to find the requested information although the participants themselves frequently commented during testing that it was easy for them to find it [48]. In a different study, patients with rheumatic diseases were enthusiastic about the possibilities of interactive apps such as peer support forums and online consultations; however, nearly all participants experienced difficulties and were not able to complete all the usability evaluation tasks while interacting with the system [49].

As in our study, other researchers and designers have underlined the importance of an iterative approach in designing mHealth apps to understand the needs of end users as well as improve app usability and feasibility [36,50]. The importance of performing usability studies on mHealth apps to be used in a clinical and patient setting therefore needs serious attention. User testing is an essential part of developing mHealth apps, especially when aiming to effectively change actual patient behavior and/or affect patient outcomes.

### Strengths and Limitations

A limitation is that the TA sessions took place in a laboratory setting. In their own home, participants may have taken more time to take a look at the app again. One of the strengths of this study is that the sample size is adequate for obtaining usability problems and that we used a mixed-methods approach— we combined the results of a TA test with the results of questionnaires on demographics, user characteristics, SUS, perceived value (IMI), and evaluation of the app. Another strength of our study is that it was performed by a multidisciplinary team and that the TA study is part of a process in developing an mHealth app, which started with 2 multidisciplinary focus group meetings [29].

Due to a lack of variety in HL levels, we were unable to analyze its potential influence on the TA outcomes. However, the recruitment of only 1 out of 12 participants with limited HL is in line with the estimations of HL prevalence levels in the Netherlands [51]; this certainly applies to a working population.

It is possible that the intention to follow the work advice could change according to the end user’s job. However, as a significant proportion of the participants was not able to open the work advice page in the app, and/or understand the work advice or intend to follow it, we think that the influence of profession is limited in this study. For the next study, we would advise asking participants about their job.

To human factor specialists, it is well known that end users should be involved from the beginning when developing an mHealth app. However, those who are well informed about a particular health domain, but less so about medical informatics, should be aware that an iterative multidisciplinary approach with the involvement of the target group from the start by using UEM research in the project is essential and can be very valuable.

The mixed-methods approach provides an insight into the cognitive process of a specific user group—pregnant working women—and their intention to use the P and W app. The TA results, in combination with the questionnaires on the perceived usability and value and the evaluation of the app, showed that incorrect interpretation of terminologies in the system prevented the end users from receiving the correct work advice. They also experienced problems with understanding the work advice because of central design problems in the interface. Despite many usability problems, the participants were relatively positive about the P and W app; the information provided in the app is considered valuable to the end users and meets their needs. The usability findings of this research could then be used to drive recommendations for developers for the next iteration of the P and W app aimed at pregnant working women.

### Conclusions

The overall conclusion of this study is that the information provided in the P and W app was considered valuable to the end users, working pregnant women, and meets their needs; however, the usability issues severely impacted the perceived usefulness of the work advice given in the app. The results of this study draw attention to the relation between effective health apps and how their design might hamper their effectiveness in changing patients’ behavior. An iterative UEM multidisciplinary approach, with the involvement of the target group from the beginning, is therefore essential for the development of health apps.

The mHealth app will be redesigned and tested in an intervention study, a survey on the effect of the app on actual work adjustment by pregnant women. A future version of the P and W app will be a valuable tool for informing pregnant women about pregnancy-related work risks.

## Appendix

Multimedia Appendix 1 Questionnaire before think aloud sessions & NVS-D.

Multimedia Appendix 2 Think aloud session protocol.

Multimedia Appendix 3 Participant tasks during the think aloud sessions: description, achievement and inclusion motivation.

Multimedia Appendix 4 Questionnaires after think aloud session: SUS, IMI, and questionnaire 3.

Multimedia Appendix 5 Overview of all usability problems and bugs.

## Abbreviations

HL: health literacy
IMI: Intrinsic Motivation Inventory
mHealth: mobile health
P and W app: Pregnancy and Work app
SUS: System Usability Scale
TA: think aloud
UEM: usability evaluation method
