# Supplementary material for: Usability and Usefulness of a Mobile Health App for Pregnancy-Related Work Advice: Mixed-Methods Approach
Source: JMIR Mhealth Uhealth. 2019 May 9;7(5):e11442. doi: 10.2196/11442 (PMC6532337; doi:10.2196/11442)
Supplement: Multimedia Appendix 1 [file mhealth_v7i5e11442_app1.pdf]

Appendix I – Questionnaire before Think Aloud sessions & NVS-D

(NVS-D can be requested)

Beste mevrouw,

Hartelijk dank voor uw interesse in dit onderzoek. Voordat wij u kunnen uitnodigen voor het testen van de 'Zwangerschap en Werk' App, willen wij u een aantal vragen laten invullen. We gebruiken deze vragen om deelnemers in groepen te kunnen indelen.

Uw antwoorden worden geanonimiseerd en enkel gebruikt voor wetenschappelijke doeleinden.

Wanneer u wilt stoppen met deze vragenlijst, mag u dit te allen tijde doen. U bent niet verplicht om een reden te geven waarom u wilt stoppen.

Indien u verdere vragen heeft kunt u deze aan de onderzoekster vragen.

Bij voorbaat dank.

Met vriendelijke groet,
Het onderzoeksteam

Vragenlijst

1) Hoeveel weken bent u zwanger?

...... weken

2) Heeft u een baan?

(Vink aan wat voor u van toepassing is)

☐ Ja
☐ Nee

3) Bent u in het bezit van een mobiele telefoon?

(Vink aan wat voor u van toepassing is)

☐ Nee (ga door naar de *Opdracht*)
☐ Ja, een mobiele telefoon met internet (iPhone / smartphone)
☐ Ja, een mobiele telefoon (bijvoorbeeld een Nokia)

4) Hoe vaak gebruikt u uw mobiele telefoon?

(Vink aan wat voor u van toepassing is)

☐ Bijna nooit, alleen om bereikbaar te zijn
☐ 1x per dag of minder
☐ Meerdere uren per dag

Assignment and questionnaire NVS-D can be requested

Contact

Indien u mee mag doen aan het onderzoek, hoe kunnen wij u het beste bereiken?

Naam:

……………………………………………………….

☐ **Emailadres:**

……………………………………………………….

☐ **Telefoonnummer:**

……………………………………………………….

Einde van de vragenlijst.
