# Supplementary material for: Usability and Usefulness of a Mobile Health App for Pregnancy-Related Work Advice: Mixed-Methods Approach
Source: JMIR Mhealth Uhealth. 2019 May 9;7(5):e11442. doi: 10.2196/11442 (PMC6532337; doi:10.2196/11442)
Supplement: Multimedia Appendix 2 [file mhealth_v7i5e11442_app2.pdf]

Multimedia Appendix 2 – Think Aloud session protocol

Protocol TA Sessions 'Zwangerschap en Werk' App

Researcher: L. A. van den Berg
Participants: 5-8 per HL group; ≥ 15 participants
Duration: Estimated +/- 30 minutes

Preparation:

1. Check/print enough informed consent forms.
2. Check/print enough HL forms and post TA session questionnaires.
3. Check if the correct equipment is set up and functions accordingly:
   - ☐ Laptop
   - ☐ Camera (on tripod)
   - ☐ Pen
   - ☐ Power Bank
4. Check if there is enough battery life and memory space on equipment:
   - ☐ Laptop
   - ☐ Camera (on tripod)
5. Print out list of possible participants for that day.
6. Start recruiting participants. Ask potential participants whether they match the inclusion criteria (pregnant <20 weeks, working, native speaker) and whether they are willing to participate.

HL assessment:

7. Explain the procedure of the session. "Thank you for participating in the research. Within this research we are going to look at the user friendliness of a new app, the 'zwangerschap en werk' app. During this sessions I will give you a number of questionnaires and I will let you test the app on your own telephone. When you'll test the app on your phone I'll record the screen of the phone with this camera. Is everything clear? Do you have any questions? Great, then I would like to ask you to fill in this informed consent first."
8. Give **informed consent form** and let the participant fill it in.
9. "We will start with a short list of questions. On this page you'll find a food label. The questions on the other page are all referring to the food label. Fill in all the questions and take as long as you like".
10. Give the **NVS-D**.
    - ☐ NVS-D (duration: 3-5 minutes)
11. If the participants asks to use a calculator, she is allowed to do so. But you cannot offer a calculator without her asking.

TA session:

12. "Now we will start testing the application on your own phone. We are going to move to this part of the table where you can see a square marked with tape." Coordinate the participant to the camera set-up.
13. "Before we start the actual testing of the app I am going to explain how the procedure is going to work. You are going to do a so-called Think Aloud test. During this test you need to keep talking about what you are doing and thing whilst using the app." Provide participant with example on the researcher's phone. "So before we start we are going to practice this with a small task. Can you show what the weather will be for tomorrow on your phone and whilst doing that keep talking? Let's give it a try."
14. Start practice round TA session. "Well done."
15. "We are almost ready to start our session. During this session I will give you a number of task to do before and in the app. When you are testing the app, let me know what you see and what you find interesting. It is important that you keep your phone as close to the table as possible and within the taped square. Any questions?"

16. Press start on camera.
17. Let participant perform the following tasks:
    - ☐ Create an account (provide user with study number).
    - ☐ Fill in the (<20 weeks) questionnaire.
    - ☐ Adjust questionnaire.
    - ☐ Find "rights and how to discuss these with your employee" page
    - ☐ Find baby messages
    - ☐ Recover (personal) advice.
    - ☐ Print your advice.
    - ☐ Go to more information page / Find the goal of the app
    - ☐ Log out
18. "Great, that's it."
19. Stop camera.

Post TA session questionnaires

20. "Okay now I'm going to ask you to fill in a few more questionnaires and that's the final part of this research. Do this as carefully and truthfully as possible."
21. Give participant the following questionnaires:
    - ☐ SUS (duration: 2 min)
    - ☐ IMI (duration: 1 min)
    - ☐ Characteristics/Miscellaneous (duration: 2 min)
22. "That was it. Thank you for participating and have a nice day."
23. Give the participant the VVV gift certificate and show her the way out.

24. Import video file to laptop and delete file on memory card.
