# Supplementary material for: Usability and Usefulness of a Mobile Health App for Pregnancy-Related Work Advice: Mixed-Methods Approach
Source: JMIR Mhealth Uhealth. 2019 May 9;7(5):e11442. doi: 10.2196/11442 (PMC6532337; doi:10.2196/11442)
Supplement: Multimedia Appendix 3 [file mhealth_v7i5e11442_app3.pdf]

Appendix III – Participant tasks Think Aloud: description, achievement, and inclusion motivation

	Task	Achieved when	Inclusion motivation
**1**	Create an account.	Participant successfully created an account and can therefore utilise the app.	In order for the end-user to utilise the app she needs to be able to make an account.
**2**	Fill in the questionnaire.	Participant successfully filled in the two pages of questionnaires (mandatory) and gains access to the home page.	In order to use the app and receive work advice, the end-user needs to fill in the questionnaire.
**3**	Adjust answers questionnaire.	Participant was able to adjust one of the answers in the aforementioned questionnaire.	The work advice provided in the Z&W app is based on the answers given in the questionnaires. If there is a change in either of these answers the user should be able to easily adjust her answers.
**4**	Find 'Your rights and tips for consultation' page.	Successfully found the 'Your rights and tips for consultation' page.	The 'Your rights and tips for consultation' page provides, what is considered by the project supervisor, essential information.
**5**	Find 'baby message(s)'.	Either located the 'baby message' on the home page or finds the 'All baby messages' page.	The baby messages were added by the developers to stimulate recurrent visits by the user.
**6**	Find the 'Your work advice' page.	Participant reached the 'Your work advice' page.	The main aim of the Z&W app is to provide end users with pregnancy related work advice. Finding this page is therefore vital for achieving this goal.
**7**	Find the 'PRINT/SAVE' button.	Participant located the 'PRINT/SAVE' button on the 'Your work advice' page.	The end-user should be able to print and save their work advice. This documentation can potentially be used for future discussion with an employer or care giver.
**8**	Find the goal of the Z&W app.	Participant located the goal of the app on the 'About us' or 'About this app' page.	End-users will not use the app in a research setting, where the goal is explained beforehand. The end-user should therefore be able to locate the goal of the app on the app itself.
**9**	Log out of the app.	Participant successfully logged out of the app.	Whether the participant can log out was added to simulate a normal session and to check whether users could find the log out button.
