# Supplementary material for: Usability and Usefulness of a Mobile Health App for Pregnancy-Related Work Advice: Mixed-Methods Approach
Source: JMIR Mhealth Uhealth. 2019 May 9;7(5):e11442. doi: 10.2196/11442 (PMC6532337; doi:10.2196/11442)
Supplement: Multimedia Appendix 4 [file mhealth_v7i5e11442_app4.pdf]

Appendix IV Questionnaires after Think Aloud session

Appendix IV-I Questionnaire 1 (SUS= System Usability Scale)

Vragenlijst 1

Alle onderstaande stellingen gaan over de app die u zojuist getest heeft. Geef aan in hoeverre u het eens bent met de volgende stellingen.

1. Ik denk dat ik deze app graag regelmatig wil gebruiken.

Sterk mee oneens				Sterk mee eens
1	2	3	4	5

2. Ik vond de app onnodig complex.

Sterk mee oneens				Sterk mee eens
1	2	3	4	5

3. Ik vond de app makkelijk te gebruiken.

Sterk mee oneens				Sterk mee eens
1	2	3	4	5

4. Ik denk dat ik ondersteuning nodig heb van een technisch persoon om deze app te kunnen gebruiken.

Sterk mee oneens				Sterk mee eens
1	2	3	4	5

5. Ik vond dat de verschillende functies in deze app erg goed geïntegreerd zijn.

Sterk mee oneens				Sterk mee eens
1	2	3	4	5

6. Ik vond dat er teveel tegenstrijdigheden in de app zaten.

Sterk mee oneens				Sterk mee eens
1	2	3	4	5

7. Ik kan me voorstellen dat de meeste mensen zeer snel leren om deze app te

Sterk mee oneens				Sterk mee eens
1	2	3	4	5

gebruiken.

8. Ik vond de app erg omslachtig in gebruik.

**Sterk mee oneens**				**Sterk mee eens**
1	2	3	4	5

9. Ik voelde me erg vertrouwd met de app.

**Sterk mee oneens**				**Sterk mee eens**
1	2	3	4	5

10. Ik moest erg veel leren voordat ik aan de gang kon gaan met deze app.

**Sterk mee oneens**				**Sterk mee eens**
1	2	3	4	5

Appendix IV-II Questionnaire 1 (IMI= Intrinsic Motivation Inventory)

Vragenlijst 2

Alle onderstaande stellingen gaan over de app die u zojuist getest heeft. Geef hieronder aan in hoeverre de volgende stellingen bij u van toepassing zijn.

1. Ik geloof dat deze app mij iets zou kunnen opleveren.

Helemaal niet waar		Een beetje waar				Helemaal waar
1	2	3	4	5	6	7

2. Ik denk dat het gebruik van de app nuttig is.

Helemaal niet waar		Een beetje waar				Helemaal waar
1	2	3	4	5	6	7

3. Ik denk dat het gebruik van deze app belangrijk is.

Helemaal niet waar		Een beetje waar				Helemaal waar
1	2	3	4	5	6	7

4. Ik zou bereid zijn om de app opnieuw te gebruiken, omdat de app iets van waarde voor mij heeft.

Helemaal niet waar		Een beetje waar				Helemaal waar
1	2	3	4	5	6	7

5. Ik denk dat het gebruik van deze app mij ergens in helpt.

Helemaal niet waar		Een beetje waar				Helemaal waar
1	2	3	4	5	6	7

6. Ik denk dat het gebruik van deze app gunstig is voor mij.

Helemaal niet waar		Een beetje waar				Helemaal waar
1	2	3	4	5	6	7

7. Ik denk dat deze app belangrijk is.

Helemaal niet waar		Een beetje waar				Helemaal waar
1	2	3	4	5	6	7

Appendix IV-III Questionnaire 3

Vragenlijst 3

Dit is de laatste vragenlijst. Hieronder staan wat vragen over uzelf. Zoals eerder al was vermeld, deze informatie zal met niemand gedeeld worden.

Vraag 1. Heeft u van de app werkadvies gekregen?
(Vink aan wat voor u van toepassing is)

☐ Ja (ga door naar vraag 2)
☐ Nee (ga door naar vraag 3)

Vraag 2. Zo ja, bent u van plan iets met dit werkadvies te doen?
(Vink aan wat voor u van toepassing is)

☐ Ja
☐ Nee

Vraag 3. Hoe oud bent u?
......

Vraag 4. Bent u eerder zwanger geweest?
(Vink aan wat voor u van toepassing is)

☐ Ja
☐ Nee

Vraag 5. Heeft u al kinderen?
(Vink aan wat voor u van toepassing is)

☐ Ja
☐ Nee

Vraag 6. Wat is uw hoogst genoten opleiding? (wat is de hoogste opleiding die u heeft afgemaakt)
(Vink aan wat voor u van toepassing is)

☐ Basisschool
☐ VMBO
☐ HAVO
☐ VWO
☐ MBO
☐ HBO
☐ WO
☐ Anders, namelijk: ....

Vraag 7. Hoeveel uur werkt u gemiddeld per week?

..... uur

Vraag 8. Van welke van de onderstaande apps maakt u wel eens gebruik?
(Vink aan wat voor u van toepassing is en omcirkel hoe vaak u deze app gebruikt.)

☐ WhatsApp meerdere keren per dag / dagelijks / wekelijks / maandelijks
☐ Facebook meerdere keren per dag / dagelijks / wekelijks / maandelijks
☐ Instagram meerdere keren per dag / dagelijks / wekelijks / maandelijks
☐ Twitter meerdere keren per dag / dagelijks / wekelijks / maandelijks
☐ Snapchat meerdere keren per dag / dagelijks / wekelijks / maandelijks
☐ YouTube meerdere keren per dag / dagelijks / wekelijks / maandelijks
☐ Pokémon GO meerdere keren per dag / dagelijks / wekelijks / maandelijks
☐ Google Maps meerdere keren per dag / dagelijks / wekelijks / maandelijks
☐ App voor uw e-mails meerdere keren per dag / dagelijks / wekelijks / maandelijks
☐ Geen van de bovenstaande apps meerdere keren per dag / dagelijks / wekelijks / maandelijks

Vraag 9. Maakt u gebruik van 'zwangerschap' apps op de telefoon of tablet? (Voorbeelden: Prénatal App, Zwanger en Zo, Pregnancy Tracker, etc.)
☐ Ja
☐ Nee

Vraag 10. Wat voor een cijfer zou u de app geven?
(Vink aan wat voor u van toepassing is)

☐	☐	☐	☐	☐	☐	☐	☐	☐	☐
1	2	3	4	5	6	7	8	9	10

Einde van de vragenlijst.
