# Supplementary material for: Usability and Usefulness of a Mobile Health App for Pregnancy-Related Work Advice: Mixed-Methods Approach
Source: JMIR Mhealth Uhealth. 2019 May 9;7(5):e11442. doi: 10.2196/11442 (PMC6532337; doi:10.2196/11442)
Supplement: Multimedia Appendix 5 [file mhealth_v7i5e11442_app5.pdf]

#	Problem
1	**System Feedback vs. mental model**<br>Participant vs system designer, functionality [INLOGGEN] button misunderstood as create account. The system
2	**Mismatch mental model**<br>Participant vs system designer, functionality [ACCOUNT AANMAKEN] button misunderstood. User expects a
7	**Password feedback pop-up unclear.**<br>(location on screen not visible for participant, after filling in passwords, terminology in English).
8	**Password feedback pop up unclear.**<br>(unclear whether she needs to press on it or related to strength of password)
9	**Password feedback pop up unclear.**<br>(unclear for that feedback message 'mismatch' appears in 'real-time', while typing second password. This confuses users.)
10	**Password feedback pop up unclear.**<br>(lack of guidelines)
12	**Password feedback pop up**<br>(Feedback makes user uncomfortable.)
13	**System feedback - incorrect use of Dutch terminology and lack of adequate information how to react on the**
14	**Layout problem month button / inefficient navigation**
15	**System Feedback issue**
16	**Functionality**<br>(Month selection functionality on OS iPhone interfers with app's functionality.)
17	**Terminology problem**<br>(English)
19	**Mismatch mental model / functionality**
20	**Mismatch mental model / Inefficient feedback**<br>(User does not know whether an account has been made)
21	**Mismatch mental model**<br>(Previous experience expectancy issue)

22	**Feedback issue**
23	**Participant feedback - frustration**
24	**Readability & Layout**
25	**Screen layout & proximity**<br>Layout 'verstuur' + 'vorige' buttons
26	**Readability**<br>**(letters too small)**
27	**Screen layout & proximity**<br>(proximity radio buttons too small)
28	**Screen layout - button previous pregnancy not clearly visible**
29	**Terminology (interpretation)**<br>Problem with regard to question #1 related to 'vorige zwangerschap'.
30A	**Terminology (interpretation) (A)**<br>Problem with regard to question #1.1 related to 'medisch probleem (vorige zwangerschap)'.
30B	**Terminology (Consequence of A)**<br>(content missing option) Q #1.1
31	**Terminology (interpretation)**<br>Problem with regard to question #1.1 related to 'medisch probleem (doodgeboren/overleden kindje)'.
32	**Terminology (interpretation)**<br>Problem with regard to question #2 related to 'medisch probleem (vorige zwangerschap)'.
33	**Terminolgy (interpretation)**<br>problem with regard to question #2 related to 'klachten gezondheid'.
34	**Readability & reading strategy**<br>Only reads first section of question #4 and therefore might not answer correctly.
35	**Readability & reading strategy**<br>Question #5 appaears to be confusing for user.
36	**Readability & reading strategy**<br>Only reads first section of question #5 and therefore might not answer correctly.
37	**Terminolgy (interpretation)**Question #6
38	**Terminology / readability question #7**<br>(Two questions in one, and negation question)
39	**Terminolgy (interpretation)**<br>(Problem with regard to question #8 related to 'geweld'.
40	**Terminology (interpretation)**<br>Problem with regard to question #9 related to 'chemische stoffen'. Whether an unmentioned substance is considered a chemical solution.

42	**Readability**.<br>[illegible]
43	**Terminoloy (interpretation)**<br>Question #10
44	**Terminology problem (interpretation)**<br>with regard to question #11 related to 'Ultrageluid'.
45	**Terminolgy (interpretation)**Question #11 related to [Hitte &
46	**Terminolgy (interpretation)**<br>problem with regard to question #11 related to 'overdruk', 'lichaamstrillingen', and potentialy 'ultrageluid'.
48	**Terminology issue**<br>(English)
49	**Mismatch mental model**<br>(Understandability)
50	**Mismatch mental model / Layout problem**
51	**Mismatch mental model**<br>(Inefficiency in locating rechten and tips due to unexpected
53	**Text does not fit within the screen's layout.**
55	**Mismatch mental model.**<br>Participant vs system designer, functionality [Uw werkadvies] button unclear and not located by user in relation to werkadvies.
56A	**Mismatch mental model. (A)**<br>Participant vs system designer, functionality [Uw werkadvies] unclear and text of home screen is interpreted as work advice.
57	**Textual description appears twice**
58	**Missing content / Mismatch mental model**<br>Expectancy issue related to content.
59	**Mismatch mental model.**
61	**Mismatch mental model**<br>Expectancy issue- system does not fit user expectation.,
62	**Mismatch mental model**<br>(expactancy) / bug
63	**Readability / terminology**

64	**Insufficient feedback / navigation unclear**<br>(User user does not notice she is missing information due to lack of direct system feedback on current state)
65	**Emotional reaction to information / interpretation**
66	**Mismatch mental model**<br>(Functionality / Navigation of HOME button)
69	**Mismatch mental model**<br>(Expactancy issues related to functionality and navigation of [HOME] button).
70	**Mismatch mental model.**<br>(Expactancy issues related to navigation of [HOME] button)
71	**System feedback vs. mental model**<br>**Error in interpretation due to incorrect date fill in in questionnaire**
72	**Layout / Readability**
73	**Mismatch mental model**<br>Functionality / expectancy
74	**Readability**<br>User does not feel heard/taken seriously
76A	**Mismatch mental model**(Navigation)
77	**Mismatch mental model**<br>(expectanyc issue with regard to adjustment of questionnaire)
79	**Inconsistency in information presentation on work advice.**
80	**mismatch mental model**<br>(no perceived work advice)
81	**Textual description is recurrent.**<br>User does not feel personally adviced.
82	**Textual description is recurrent**<br>User feels overloaded.
83	**Missing content / expectancy content mismatch**<br>Expectancy issue related to content.

84	**Mismatch mental model & readability**<br>Readability / Understandability<br>(Text indicates that all options were chosen on question #1.1)
85	**Terminology**<br>(Interpretation title: Vrijheid)
86	**Terminology**<br>(Interpretation title: Geen)
87	**Layout**<br>(Confusing layout)
88	**User preferences**
89	**Emotional reaction to information/ interpretation**
90	**Mismatch mental model.**<br>Expectancy issue related to printing funcitonality.
91	**Readability & Layout of the screen**
92	**Inefficiency in locating goal of the app due to unexpected location on screen**
93	**Mismatch mental model & navigation**
94	**Terminology**
95	**Navigation**
97	**Mismatch mental model & navigation**
98	**Mismatch mental model & Layout [stop deelname]**

B1	**Bug**
B2	**Bug**
B3	**Bug**
B4	**Bug**
B5	**Bug**
B6	**Bug**
B7	**Bug**
B8	**Bug**
B9	**Bug**

B10	**Bug**
B11	**Bug**

Description	3	4	5	6	7
Fills in a username and password and then [INLOGGEN]. Assumes that with these entries she'll get an account. [incorrect expection related to creating an account). Note that There might have been confusion with regard to the			X		
Fills in a username and password and then [ACCOUNT AANMAKEN]. Assumes that with these entries she'll get an account.			X		
[Bad] pops up because password does not conform to (unexplained) guidelines. Participant expects something different. Thinks it means that she hasn't properly filled in all the entries. Later attributes [Bad] to not having a good password (02:21). Rectifies/understands her 'mistake' with adding an extra character (02:33).	X				
[Good] pops up. User taps it multiple times, but nothing happens. Expresses her confusion as to whether she needs to press it or if it means that she entered a 'good' password.				X	
While filling in her second password [mismatch] pops up. User does not understand that this feedback is 'real-time' (i.e. if you have only typed in 2 letters then there's a 'mismatch'). This results in her retyping in her passwords a number of times.				X	
[Bad] pops up because password does not conform to (unexplained) guidelines. User does not know / notice and proceeds. Other user					X
[Mismatch] pops up during typing the second password. User says that the pop-up alerts/scares her.					
This message pops up after entering a [bad] password. Which seems a little too late. One would assume this would be provided during the first time					X
Cannot immediately find the [Apr] button to select the month. Had to scroll up.	X	X	X	X	X
Had to scroll up to select month. Tapped the arrow to the right. App scrolled down automatically. User had to scroll up again.			!		
Calendar (buttons) too small, user accidentally taps field behind the calendar.					
User, again, searches for the month. But this time around she does find it. Which results in her completing all the entries on this page.					
User first types in the current date (day). Then hovers her finger over the calendar (potentially trying to find the month button). Taps 2017, nothing happens. Closes the calendar to type the date in herself. She then uses -					
User expects to select the month when she taps [Sep]. However, nothing happens. She needs to 'confirm' this selection through a OS mechanism where she needs to press [Done].					
The 'I have read and agree' is in written in English. The user comments that her English is not very good. She then does not select this section. Therefore, the [CONTINUE] button will not pop-up.					
User notices English words and phrases: 'I have read and agree' + 'Terms and Conditions' + 'Continue'.					
Because the user didn't complete/fill in all the question the [VOORTZETTEN/CONTINUE] button does not appear. Because the button does not appear the user is unaware such a button even exists. She finds the					
Does not know whether she has made an account. (After, for whatever reason, failing on the Account aanmaken page). Tries to log in to test whether she has an account. Fails.				X	X
Doubts about tapping [ACCOUNT AANMAKEN], because it did not function the last time. Taps it again provides feedback with regard to [bad] password. She understands this immediately and fixes it at 04:26.					X

User does not know why she was unable to make an account the previous time (did not complete the ACCOUNT AANMAKEN page). She talks about her password strength, but comments that she was told that it was good enough.					
User expresses her frustration for being, so far, unable to have made an account.					
User feels that the different headings above the questions are redundant. Needs to do a double take in order to understand the questions.					
The [VORIGE] button is (with the same shape and color) the first button, above the [VERSTUUR] button. User LBTA010 accidentally presses [VORIGE].					
User almost presses [VORIGE] instead of [VERSTUUR]. She comments on how she expected the [VERSTUUR] button on top.					
User comments that she find the letters in the questionnaire page too small.				X	
User means to select [Nee], taps [Ja]. Radio buttons perhaps to close together.				X	
Text states it's about her previous pregnancy, but she hasn't had a previous pregnancy. User expresses her confusion. Takes a few seconds to find the 'dit is mijn eerste zwangerschap' option.		X			
The participant is unsure whether the 'vorige zwangerschap' implies her previous pregnangy, not those which have come before that. She assumes the it implies her direct previous pregnancy.	X				
Previous pregnancy was terminated because of a medical condition. User does not know how this fits within the given options. She is doubting between two options: 'overleden kindje' & 'kindje geboren voor 37 weken zwangerschap duur'.			X		
Related to the previous usability problem: Expects another option to be included which is: 'miskraam'			X		
User expresses some doubts about the meaning/distinction between [Overleden kindje] and [Doodgeboren].					
User is confused/conflicted whether this is about her health status before her previous or current pregnancy.			X		
User clicks [Ja] on question #2 to see what the options are. This implies that the question itself wasn't clear enough for the participant.					
Reads first part of the question aloud and already answers the question without reading the details of the question. Comments that she does not read the full text.	X				
Two questions in one. One user reads first part of the text, then the last part, then the middle part. Other user remarks that there are two questions in one.	X				
Reads first part of the question aloud and already answers the question without fully reading. The cause of this action is unclear (e.g., there is not too much text). It is clear that she purely answers the first part of the question (>40 hrs / week). If she works night shifts which we don't know) she would have incorrectly filled in this question.				X	
User comments on question #6 and how one can interpret it.					X
Users struggles with the two questions put into one. Afterwards struggles with the negative nature of the question. Has to review the question and adjust her answer.					X
Has some slight doubt but in the end is pretty certain about her answer.					
The user is unsure whether lachgas is considered a chemical solution.					

The user is not completely sure whether hair dye is a chemical solution.					
Again, reads only first part of the question (not the details) and answers question based on the first part	X			X	
Reads first part of the question aloud and then stops talking for a little while. She may or may not have read the details of this question				!	
Says she comes in contact with 'rauw vlees' sometimes (which is one of the examples given), but selects [Nee].					X
Questions what 'ultrageluid' is.			X		
User comments on question #11 and how one can interpret the [Hitte & Kou] option.					X
It is unclear to the user what is meant by overdruk and lichaamstrillingen. She belives that lichaamstrillingen are shivers. She probably also misintepreted ultrageluid as loud noise.					
Participant remarks that the weeks and days are in English.	X				
Expects changes on home page after adjusting her questionnaire. User expresses she expected changes in baby bericht section.	X				
Is confused about whether she can tap [Uw werkadvies] or whether it is the title of the text underneath.			X		
Expects 'Uw rechten en tips voor overleg' in the text on top of the home page. One participant proceeds to click on URL to the RIVM page. The other					
Parts of text do not 'fit' on the screen. One participant comments on how she needs to scroll to the right to be able to read the text. (Uw rechten en tips voor overleg page). The others do not comment on it but do experience the	X	X	X	X	X
Appears to believe that she has not received work advice. (Will be directed to it at 11:07).	X				
User does not interact with this button and proceeds to look for work advice elsewhere on the app.					
Believes that her workadvice is depicted on home page. Does not utilise the [Uw werkadvies] button, therefore completely misses the work advice page. This gets confirmed at 16:40					
User notices that two pieces of text on the home page are identical.				X	
Expects some mentioning of the 'vijfde ziekte' in the text.				X	
Expects tips surrounding the baby in/on the 'baby bericht' page.	X				
The participant is 10 weeks pregnant, however the baby bericht showcases a text about a baby that is 8 weeks old. User expects baby bericht about a 10 week old baby.		X			
User is more weeks pregnant than what the baby bericht is about. User expresses her confusion about whether the system got it wrong and why she isn't getting information about 19 weeks.					
[Lees verder] does not result in more information, just a new page. Participant states that she finds this odd. She expected more information.					
User states that she will not watch the video because it isn't clear what the video is about. She says if there were a title or some explanation she might watch it.					

User taps [Volgende] and does not notice (the first time) that she's on a new page. When page loads, it isn't clear that it's loading. It also doesn't go to the top of the page. So the user believes she is still on the same page.<br>This made her miss information of one entire page.				X	
Text indicates that the fetus should be around a certain length. User just had an ultrasound and sounds slightly worried that her child was slightly smaller than the text indicates.				X	
Has a belief that when tapping [HOME] she will be logged out. This does not happen, so she learned that this is not the case.	X				
User believes that she can adjust her settings on the home page. She also does not notice that she is already on the home page.				X	
The home page reloads. User was not aware she was already on the home page.				X	X
The user comments that she isn't 12 weeks and 3 days pregnant (11 weeks). This problem is not the same as usability problem #61. Within this problem the user apparently did not correctly fill in her [Uitgerekende datum]. The app is functioning fine, but the participant is misinformed about how many weeks she is pregnant or she made a mistake when filling in the [Uitgerekende datum]. If the latter there might be an issue with regard to filling in the [Uitgerekende datum] if it is this easy to make an mistake. Additionally, it is impossible to adjust the [Uitgerekende datum]. So when a mistake is made cannot be corrected.					
User says that there's too much text.					
User expects URLs to be underlined.					
User does not feel taken seriously. Text replies to a question. ('Heb je geprobeerd aan je stress te doen? Goed zo')					
User wants to go back to the previous page instead of searching for a [Uw vragenlijst aanpassen] button.					X
User believes that all her previous entries are saved in the system. She thinks that if she were to go to the next page (without filling in all the entries), she could just continue. She clicks the button and a feedback message					
The app indicates that there is no work advice. However, when scrolling down the page she does receive some work advice with regard to her previous pregnancy (12:02).	X				
The app indicates that there is no work advice. User says: "My work advice, none. Oh well that's easy. It says I don't need to adjust anything.". However, when scrolling down the page she does receive some work advice with regard to her previous pregnancy (12:02). She mentions these work advices. Later on in the questionnaire she states that she did not receive work advice.	X				
Certain bits and pieces of the categories overlap. Advice is repeated a number of times. Results in not fully reading the full advice. She does not expect new information.	X				
Certain texts in the werkadvies and home page text overlap. Advice is the repeated a number of times. Results in not fully reading the full advice. She does not expect new information.				X	
Expects more information about 'Sommige infecties kunnen schadelijk zijn..'. This wish was also mentioned earlier in this think aloud session.				X	

Text indicates that the user has suffered from ALL the '#1.1 medisch probleem - vorige zwangerschap' options. When in fact she has only chosen 1 (Overleden kindje), which is only noticable via the title of this paragraph. User expresses her confusion.			X		
User says that she doesn't like the title. She says she doesn't really know what is meant by it and would normally scroll past it.					
User doesn't know what is meant by the title of the text.					
User expresses that she finds the layout weird. Her screen shows titles that don't have subtext because she didn't have any complaints, etc. She doesn't understand this.					
Expresses how she would let certain content (nachtdiensten niet mag tot na 6 maanden na de bevalling) stand out a bit more.					
Participant tells how IVF & miskramen are in the past and scrolls past them.				X	
Participant indicates that she wouldn't easily print something of her phone. Is curious and reluctant as to whether the PRINTing functionality would work.	X				
User comments that there is a lot of text on the 'Over de app' page.	X				
User expresses that such information (goal of the app) can be found mostly on the home page. This is not the case.					
Cannot find more information about the goal of the app. Potentially believes that there should be a distinct section/button titled: 'Goal of the App'					
Inconsistency with regard to form of address		X			
User says she does not know which page she will go back to if she presses the [TERUG] button. It takes her back to literally the previous page.					
The [TERUG] button literally goes back to the previous page. The user (seemingly) suspects that it will take her back to her previous page in the order she visited them (like in a browser). This gets her stuck in a loop for a number of tries, until she uses the [terug] button in the menu on the page itself.					
User nearly presses the [STOP DEELNAME] button because it stands out from the other buttons.					

The 'Hulp nodig?' page has not content (yet).					
Taps [VOORTZETTEN], nothing happens. Presses the [Log In] button, because of the bug she cannot continue (VOORTZETTEN).				X	X
When user clicks on link [Wet- en regelgeving zwanger en werk] nothing happens.			X		
Taps [HOME] on home page. Loads a 'about:blank' page. User LBTA007 fixes it by re-entering the URL of the app.					X
User taps [Alle baby berichten] twice, nothing happens.			X		
User taps [HOME]. Button does turn red, but nothing happens.			X		
On homepage: ‘Sorry, Probeer Het Opnieuw Of Neem Contact Met Ons Op Via Info@Zwangerschapenwerk.nl’ (rest of app works accordingly) pops up.					
Taps [HOME] on uw werkadvies page. Loads a 'about:blank' page. User fixes it through using the return button on her phone.					
User taps [Hier staan de wettelijke regels], results in a log-in page. Because of this goes back, does not read the page, and misses its content.					

Cannot find the PDF functionality. Additionally cannot find the [TERUG] buttons etc. LBTA003 resolves this around (14:49), then the buttons are there (they were not before).	X	X			
[TERUG] button on baby bericht week 12 does not function accordingly. Participant solves it through clicking on [HOME].	X				

**Total number of usability problems**	17	5	10	18	12
**Bugs**	2	1	3	1	2
Total Usability Problems + Bugs	19	6	13	19	14
**Total number of** *mismatch mental model* problems per participant	6	1	4	3	4
**Total number of** *terminology* problems per participant	2	1	4	0	4
**Total number of terminology (domain k.) problems per participant**	1	0	4	2	4
**Average severity**	2,6	2,6	3,4	2,3	3,0

8	9	10	11	12	13	14	Location (page)	#		Severity		UTP (artifact)
	X			X			Log in	3		2		Visualness-non message feedback
X	X		X				Log in	4		2		manipulation - cognitive aspects
							Account aanmaken	1		3		language - other wording - error messages (- English)
							Account aanmaken	1		2		Visualness-non message feedback
X	X		X				Account aanmaken	4		2		Visualness-non message feedback
		X					Account aanmaken	2		2		language - other wording - error
			X				Account aanmaken	1		1		Visualness - object appearance
							Account aanmaken	1		3		Language - other wording - error
X	X	X	X	X	X	X	Account aanmaken	12		4		Manipulation - physical aspects
	!											
				!								
				X			Account aanmaken	1		4		Visualness-non message feedback
X							Account aanmaken	1		1		Not related to app
			X			X	Account aanmaken	2		4		language - naming/labeling (- English)
						!						
	X		X	X			Account aanmaken	3		4		Visualness - object appearance
	X		X	X			Log in	5		4		Visualness-non message feedback
							Account aanmaken	1		1		

				X			Account aanmaken	1	3	Visualness-non message feedback
				X			Account aanmaken	1	2	
						X	Questionnaire	1	1	Visualness- objects (screen) layout
		X				X	Questionnaire	2	2	Manipulation - physical aspects (- proximity)
						!				
							Questionnaire	1	2	Visualness- objects (screen) layout
							Questionnaire	1	2	Manipulation - physical aspects (-
							Questionnaire	1	2	Visualness- objects (screen) layout
							Questionnaire (Q1)	1	4	language - naming/labeling
							Questionnaire (Q1.1)	1	4	language - naming/labeling
							Questionnaire (Q1.1)	1	4	wording - user-requested information/results
X							Questionnaire (Q1.1)	1	4	language - naming/labeling
							Questionnaire (Q2)	1	4	language - naming/labeling
					X		Questionnaire (Q2)	1	3	language - naming/labeling
							Questionnaire (Q4)	1	2	language - other wording - on-screen text
			X				Questionnaire (Q5)	2	1	language - other wording - on-
							Questionnaire (Q5)	1	2	language - other wording - on-screen text
							Questionnaire (Q6)	1	3	language - other
							Questionnaire (Q7)	1	3	language - other wording - on-screen text
				X			Questionnaire (Q8)	1	0	
	X		X				Questionnaire (Q9)	2	4	language - naming/labeling

			!							
X		X		X			Questionnaire (Q9)	5	2	
							Questionnaire (Q10)	1	2	language - naming/labeling
					X		Questionnaire (Q11)	2	3	language - naming/labeling
							Questionnaire (Q11)	1	3	language - naming/labeling
					X		Questionnaire (Q11)	1	3	language - naming/labeling
							Home	1	1	language - naming/labeling (-English)
							Home	1	2	Visualness - non-message feedback
							Home	1	4	Visualness - object (screen) layout
		X			X		Home	2	3	Visualness - object (screen) layout
X	X	X	X	X	X	X	R & t voor overleg	12	4	Visualness - object (screen) layout
					X		Home	2	4	visualness - presentation of information / results
					!					
X					X		Home	2	4	visualness - presentation of information / results
						X	Home	2	1	language - other wording - on-
							Home	1	2	language - other wording - user-
							Home	1	1	language - other wording - user-
						X	Home	2	2	language - other wording - user-requested
						!				
						X	Home	1	2	manipulation - physical aspects
						X	Home	1	1	language - naming/labeling

		X					Alle baby berichten	2	3	visualness - non-message feedback
							Home	1	1	
							General (functions)	1	1	language - naming/labeling
							Home	1	2	manipulation - cognitive aspects - visual cues
X	X						Home	4	3	manipulation - cognitive aspects - visual cues
	X						Home	1	2	
						X	Home	1	2	visualness - presentation of
						X	Home	1	1	language - other wording - on-screen text
						X	Home	1	1	language - other wording - on-screen text
X	X				X		Home	4	4	manipulation - cognitive aspects -
	X				X		Adjust questionnaire	2	2	manipulation - cognitive aspects - visual cues
							Uw werkadvies	1	4	language - other wording - feedback messages
							Uw werkadvies	1	3	manipulation - cognitive aspects - visual cues
							Uw werkadvies	1	3	visualness - presentation of information/results
							Home / Uw werkadvies	1	3	visualness - presentation of information/results
							Uw werkadvies	1	2	language - other wording - user-

						Uw werkadvies	1	3	language - other wording - user-requested information / results
					X	Uw werkadvies	1	2	language - naming/labeling
					X	Uw werkadvies	1	3	language - naming/labeling
					X	Uw werkadvies	1	3	visualness - presentation of information/results
X						Uw werkadvies	1	1	
						Uw werkadvies	1	1	
						Miscellaneous	1	2	
						Goal of the app	1	3	visualness - presentation of
		X				Home	1	1	
X						Home	1	4	manipulation - cognitive aspects - visual cues
						General (functions)	1	1	language - naming/labeling
		X				General (functions)	1	3	manipulation - cognitive aspects - visual cues
			X			General (functions)	1	3	manipulation - cognitive aspects - visual cues
					X	Home	1	4	visualness - object appearance
			X			Log in	1	3	
						Account aanmaken	2	4	
						Home	1	3	
						Home	1	3	
						Home	1	4	
						Home	1	3	
					X	Home	1	4	
	X					Uw werkadvies	1	4	
		X				Uw werkadvies	1	3	

Print page	2	4
Alle baby berichten	1	3

AVG **2,48**

10	14	7	12	11	10	16	**Total occurences**	142
0	0	1	1	1	0	1	**Total occurences**	13
10	14	8	13	12	10	17	**Total occurences**	155
5	9	1	4	4	5	4	**Total occurences**	50
1	1	0	2	1	3	4	**Total occurences**	23
1	1	0	1	1	3	3	**Total occurences**	21
3,0	3,0	2,9	2,8	2,9	3,4	2,3		

UTP (task)		Mismatch MM	Terminology	DomainTerm	Feedback	Layout	Readability
task facilitation - keeping the user on track		X			x		
task mapping - functionality		X					
task facilitation - user action reversal					X		
task facilitation - keeping the user on track					X		
task facilitation - keeping the user on track					X		
task facilitation - user action					X		
task facilitation - emotional					X		
task facilitation - user action					X		
task mapping - functionality						X	
task mapping - functionality					X		
Not related to app		X					
task mapping			X				
task facilitation - keeping the user on track		X					
task facilitation - keeping the user on track		X			X		
task facilitation - emotional response		X					

task facilitation - keeping the user on track			X		
task mapping				X	X
task mapping - interaction				X	
					X
task mapping - interaction				X	
task mapping - functionality				X	
task mapping - functionality	X	X			
task mapping - functionality	X	X			
task mapping - functionality	X	X			
task mapping - functionality	X	X			
task mapping - functionality	X	X			
task mapping - functionality	X	X			
					X
					X
					X
	X	X			
	X	X			X
	X	X			
	X	X			

					X
		X	X		
		X	X		
		X	X		
		X	X		
		X			
	X				
	X			X	
task mapping - navigation	X			X	
				X	
task facilitation - keeping the user task on track	X				
task mapping - functionality	X				
			X		
	X				
task mapping - functionality	X				
task mapping - functionality	X				
task mapping - functionality		X	X		X

			X		
task mapping - navigation					
task facilitation - emotional	X				
task facilitation - keeping the user task on track	X				
task facilitation - keeping the user task on track	X				
	X		X		
task facilitation - user action reversal					
				X	X
	X				
					X
task mapping - navigation	X				
task mapping - functionality	X				
			X		
task mapping - functionality					
	X				
task facilitation - keeping the user task on track					
		X			

task facilitation - task/function automation	X				X
		X	X		
		X	X		
				X	
task mapping - functionality	X				
				X	X
	X			X	
task mapping - navigation	X				
		X			
task mapping - navigation					
	X				
task mapping - navigation					
	X			X	

Total problem type: Mismatch mm	Total problem type: Terminology	Total problem type: 'domain' knowledge	Total problem type: Feedback	Total problem type: Layout	Total problem type: Readability
28	20	19	13	13	12

Navigation	Functionaility	Recurrency	Missing	User pref	Par feedback	Misc.
X						
	X					

X

X

X

X

X

X

X

X

X

X

X

X

X

Total problem type: Navigation	Total problem type: Functionalit y	Total problem type: Recurrency	Total problem type: missing c.	Total problem: User pref	Total problem: Participant feedback	Total problem: Miscellena ous
5	1	3	2	1	1	2
